# The influence of corticosteroid treatment on the outcome of influenza A(H1N1pdm09)-related critical illness

**DOI:** 10.1186/s13054-016-1230-8

**Published:** 2016-03-30

**Authors:** Jesse W. Delaney, Ruxandra Pinto, Jennifer Long, François Lamontagne, Neill K. Adhikari, Anand Kumar, John C. Marshall, Deborah J. Cook, Philippe Jouvet, Niall D. Ferguson, Donald Griesdale, Lisa D. Burry, Karen E. A. Burns, Jamie Hutchison, Sangeeta Mehta, Kusum Menon, Robert A. Fowler, on behalf of the Canadian Critical Care Trials Group H1N1 Collaborative

**Affiliations:** Interdepartmental Division of Critical Care Medicine, Department of Medicine, University of Toronto, Toronto, ON Canada; Sunnybrook Hospital, Toronto, ON Canada; Centre de recherche clinique Étienne-Le Bel, Université de Sherbrooke, Sherbrooke, PQ Canada; Department of Critical Care Medicine, Sunnybrook Hospital, 2075 Bayview Avenue, Room D478, Toronto, ON M4N 3M5 Canada; Section of Critical Care Medicine, Department of Internal Medicine, Faculty of Health Sciences, College of Medicine, University of Manitoba, Winnipeg, MB Canada; Section of Infectious Diseases, Department of Internal Medicine, Faculty of Health Sciences, College of Medicine, University of Manitoba, Winnipeg, MB Canada; Department of Medical Microbiology, Faculty of Health Sciences, College of Medicine, University of Manitoba, Winnipeg, MB Canada; Department of, Faculty of Health Sciences, College of Medicine, University of Manitoba, Winnipeg, MB Canada; St. Michael’s Hospital, University of Toronto, Toronto, ON Canada; St. Joseph’s Hospital, McMaster University, Hamilton, ON Canada; CHU Sainte Justine, Université de Montréal, Montréal, PQ Canada; Department of Medicine, University of Toronto, Toronto, ON Canada; Department of Physiology, University of Toronto, Toronto, ON Canada; Division of Respirology, Department of Medicine, University Health Network and Mount Sinai Hospital, Toronto, ON Canada; Critical Care Program, Department of Medicine, University Health Network and Mount Sinai Hospital, Toronto, ON Canada; Vancouver General Hospital, University of British Columbia, Vancouver, BC Canada; Mount Sinai Hospital, University of Toronto, Toronto, ON Canada; Division of Critical Care, St. Michael’s Hospital, University of Toronto, Toronto, ON Canada; Hospital for Sick Children, University of Toronto, Toronto, ON Canada; Children’s Hospital of Eastern Ontario, University of Ottawa, Ottawa, ON Canada; Rouge Valley Health System, Scarborough, ON Canada

## Abstract

**Background:**

Patients with 2009 pandemic influenza A(H1N1pdm09)-related critical illness were frequently treated with systemic corticosteroids. While observational studies have reported significant corticosteroid-associated mortality after adjusting for baseline differences in patients treated with corticosteroids or not, corticosteroids have remained a common treatment in subsequent influenza outbreaks, including avian influenza A(H7N9). Our objective was to describe the use of corticosteroids in these patients and investigate predictors of steroid prescription and clinical outcomes, adjusting for both baseline and time-dependent factors.

**Methods:**

In an observational cohort study of adults with H1N1pdm09-related critical illness from 51 Canadian ICUs, we investigated predictors of steroid administration and outcomes of patients who received and those who did not receive corticosteroids. We adjusted for potential baseline confounding using multivariate logistic regression and propensity score analysis and adjusted for potential time-dependent confounding using marginal structural models.

**Results:**

Among 607 patients, corticosteroids were administered to 280 patients (46.1 %) at a median daily dose of 227 (interquartile range, 154–443) mg of hydrocortisone equivalents for a median of 7.0 (4.0–13.0) days. Compared with patients who did not receive corticosteroids, patients who received corticosteroids had higher hospital crude mortality (25.5 % vs 16.4 %, *p* = 0.007) and fewer ventilator-free days at 28 days (12.5 ± 10.7 vs 15.7 ± 10.1, *p* < 0.001). The odds ratio association between corticosteroid use and hospital mortality decreased from 1.85 (95 % confidence interval 1.12–3.04, *p* = 0.02) with multivariate logistic regression, to 1.71 (1.05–2.78, *p* = 0.03) after adjustment for propensity score to receive corticosteroids, to 1.52 (0.90–2.58, *p* = 0.12) after case-matching on propensity score, and to 0.96 (0.28–3.28, *p* = 0.95) using marginal structural modeling to adjust for time-dependent between-group differences.

**Conclusions:**

Corticosteroids were commonly prescribed for H1N1pdm09-related critical illness. Adjusting for only baseline between-group differences suggested a significant increased risk of death associated with corticosteroids. However, after adjusting for time-dependent differences, we found no significant association between corticosteroids and mortality. These findings highlight the challenges and importance in adjusting for baseline and time-dependent confounders when estimating clinical effects of treatments using observational studies.

**Electronic supplementary material:**

The online version of this article (doi:10.1186/s13054-016-1230-8) contains supplementary material, which is available to authorized users.

## Background

During the 2009 influenza A(H1N1; (H1N1pdm09) pandemic, the World Health Organization reported substantial influenza-related critical illness and mortality, especially among young people [1, 2], and H1N1pdm09 is now among the most common seasonal influenza strains [3]. Severe influenza-related critical illness typically manifests as viral pneumonitis and acute respiratory distress syndrome (ARDS). Despite available treatment options, including admission to the intensive care units (ICUs), neuraminidase inhibitors, and antibiotics for concomitant or secondary bacterial infections, morbidity and mortality remain high, with seasonal influenza currently estimated to result in over 500,000 deaths globally each year [4–7].

Corticosteroids have long been used among critically ill patients with ARDS or shock. They are associated with reductions in the circulating levels of proinflammatory mediators, possible improvements in gas exchange, and reduced duration of mechanical ventilation and shock [8, 9]. However, corticosteroids increase the risk of hyperglycemia, as well as neuropathy and myopathy related to critical illness, and the effect of corticosteroids on the risk of infection and survival is uncertain for critically ill patients [10–19]. Corticosteroids have been commonly prescribed for subsequent influenza outbreaks such as avian influenza A(H7N9) [20].

Across studies, approximately one-third of patients with H1N1pdm09-related critical illness have reportedly been treated with corticosteroids. While no randomized controlled trials on this topic exist, the most recent observational studies have estimated an increased risk of death among patients receiving corticosteroids [4, 5, 7, 21–28]. Observational studies in which researchers seek to estimate treatment effects, however, are often subject to confounding by indication and large imbalances in baseline characteristics. In addition, unlike a randomized trial in which baseline characteristics are typically defined before randomization and exposure to the trial intervention, in observational studies the time from baseline to initiation of the intervention is variable. Furthermore, patients’ trajectories over this time may also influence the subsequent decision to initiate corticosteroids. Thus, to most accurately estimate the influence of a treatment, both baseline and time-dependent factors until initiation of treatment should be considered. Accordingly, we analyzed a large cohort of patients with H1N1pdm09-related critical illness to describe patterns of corticosteroid use, identify predictors of steroid prescription, and investigate clinical outcomes (adjusting for baseline and time-dependent characteristics) among patients treated or not treated with corticosteroids.

## Methods

### Study design

We conducted a multicenter observational study of critically ill patients infected with H1N1pdm09, the details of which have been published previously [4, 5, 29] and are also provided in Additional file 1: Appendixes C and D. Local research ethics boards approved the study and waived the need for informed consent at each participating site. All data management and statistical analyses were conducted using SAS version 9.2 software (SAS Institute, Cary, NC, USA).

### Data collection

**Fig. 1:**
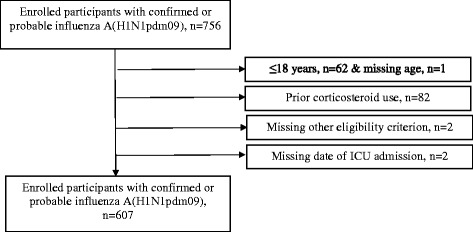
Study participant enrollment flow diagram. *ICU* intensive care unit

Data were collected from 51 sites across Canada between 16 April 2009 and 24 March 2010. Eligible patients included all critically ill adults (age >18 years) admitted to participating hospitals with confirmed, probable, or highly suspected H1N1pdm09 infection [4, 5, 30, 31]. All patients known to be receiving oral or parenteral corticosteroids before the onset of critical illness were excluded from analyses (Fig. 1).

Demographic variables, comorbidities, copresenting conditions, and severity of illness by Acute Physiology and Chronic Health Evaluation (APACHE) II score were recorded at the onset of critical illness [4, 5, 29, 32]. Time-dependent variables used to calculate the Sequential Organ Failure Assessment (SOFA) score were collected on days 1, 2, 3, 7, 14, and 28 [33]. Corticosteroid, antibiotic, neuraminidase inhibitor, or other antiviral medications were collected including type, dose, frequency, date and time of prescription as well as any change or cessation, as were secondary infections (blood, urine, and respiratory culture dates), and mechanical ventilation initiation and liberation, date of ICU and hospital admission, and discharge and vital status.

The primary outcome of this study was hospital mortality adjusting for baseline and time-dependent variables. In Canada, live discharge from the hospital is a clinically meaningful outcome because long-term acute care hospitals that provide care to seriously ill patients immediately after acute hospitalization generally do not exist. ICU mortality is important but somewhat dependent upon clinical discharge readiness decision-making. Secondary outcomes included the description of corticosteroid prescription (frequency, dose, duration, and regimen), factors associated with corticosteroid prescription, ventilator- and ICU-free days at 28 days, and the frequency of positive bacterial respiratory or bloodstream infections among patients who were treated and not treated with corticosteroids [34].

### Between-group analyses

Patients were described and analyzed in two groups on the basis of receipt of corticosteroids using a two-sample *t* test or the Wilcoxon rank-sum test for continuous variables (reported as mean [standard deviation] or median [interquartile range]), as appropriate, and using χ^2^ test or Fisher’s exact test for discrete variables (reported as number and proportion). Results from all multivariable analyses are reported as odds ratios (ORs) with 95 % confidence intervals (CIs).

### Multivariable analysis adjustment: prediction of corticosteroid use and clinical outcomes

To investigate the associations between baseline variables and corticosteroid use, a multivariable analysis was performed. The multivariable model comprised factors of clinical interest decided a priori and all significant covariates at the univariate level (*p* ≤ 0.2). Variables were entered into a multivariable logistic regression model using a forward stepwise process. We followed the generally accepted principle of including one predictor variable for every five to ten patients with the outcome of interest [35]. Relevant scatterplots and multicollinearity matrices were generated and assessed for nonlinear relations or redundant covariates, respectively. Potential redundancies were flagged where Pearson’s and Spearman’s *r* were greater than or equal to 0.2. The final list of potential or candidate variables was reconciled where necessary, based on clinical consensus. Model integrity was examined using standard diagnostic statistics and plots and goodness of fit for each model for all outcomes and was examined with the Hosmer-Lemeshow test. Two observations represented extreme points in the model and were removed from all subsequent analyses; both of these 2 patients had no corticosteroid exposure), leaving 605 patients). In a distinct multivariable analysis, we measured the associations between hospital mortality and potential risk factors, including corticosteroid exposure. We excluded 20 patients with missing outcome data, leaving a sample size of 585 patients. Because analyses to detect differences in treatment variables between survivors and nonsurvivors pose a risk of confounding due to immortal time bias (patients who die quickly have less ‘opportunity’ to be exposed to certain therapies), we performed separate sensitivity analyses in which we restricted comparisons to patients who did not die within the first 3 days after admission to the hospital, and where we adjusted for the possibility of clustering due to center effect in a hierarchical model using generalized estimating equations [36, 37].

### Propensity score adjustment

To account for residual confounding by indication of the associations between corticosteroids and clinical outcomes, we developed a propensity score to receive corticosteroid for each participant, employing 41 available covariates (Tables 1 and 2), after which SOFA score and certain laboratory values were removed as they covaried with other variables. Body mass index was removed, owing to a large number of missing values for height, and pregnant patients were removed because of small numbers and subsequent inability to match. Thus, 24 variables were included in the propensity score to receive corticosteroid model, with all patients being assigned a value between 0 and 1.

**Table 1:** Baseline characteristics of patients according to corticosteroid treatment status among critically ill patients with H1N1pdm09

Baseline characteristics^a^	All patients^b^ (*n* = 607)	Not treated with corticosteroids (*n* = 327)	Treated with corticosteroids (*n* = 280)	*p* Value^c^
Age, years	47.4 (15.3)	46.2 (15.2)	48.8 (15.3)	0.04
Female sex, *n* (%)	315 (51.9)	163 (49.9)	152 (54.3)	0.28
BMI, kg/m^2^	32.0 (10.4)	31.8 (10.7)	32.3 (10.1)	0.62
APACHE II score	20.6 (10.0)	20.1 (9.7)	21.2 (10.3)	0.22
SOFA score, day 1	11.4 (3.7)	11.3 (3.6)	11.4 (3.8)	0.70
SOFA score (cardiovascular), day 1	1.6 (1.5)	1.6 (1.5)	1.6 (1.5)	0.91
PaO_2_/FiO_2_ ratio, day 1	155.1 (92.1)	156.3 (91.8)	153.5 (92.4)	0.73
Tidal volume (ml), day 1	514.0 (131.9)	515.8 (119.5)	513.0 (144.2)	0.84
Tidal volume per ideal body weight (ml/kg), day 1	6.2 (2.1)	6.3 (2.1)	6.1 (2.1)	0.56
Positive end-expiratory pressure, (cmH_2_O), day 1	10.9 (4.6)	10.7 (4.3)	11.1 (4.9)	0.39
Patients with any comorbidities (any)	543 (89.5)	280 (85.6)	263 (93.9)	0.001
Comorbidities per patient, *n* (%)	3.2 (2.3)	2.8 (2.2)	3.7 (2.4)	<0.001
Pulmonary comorbidity, *n* (%)	195 (32.1)	67 (20.5)	128 (45.7)	<0.001
Asthma, *n* (%)	124 (20.4)	42 (12.8)	82 (29.3)	<0.001
COPD, *n* (%)	100 (16.5)	30 (9.2)	70 (25.0)	<0.001
Cardiac disease, *n* (%)	89 (14.7)	40 (12.2)	49 (17.5)	0.07
Hypertension, *n* (%)	195 (32.1)	102 (31.2)	93 (33.2)	0.60
Obesity^d^, *n* (%)	145 (23.9)	74 (22.6)	71 (25.4)	0.43
Diabetes, *n* (%)	155 (25.7)	80 (24.5)	75 (26.8)	0.51
Immune suppression^e^, *n* (%)	35 (5.8)	10 (3.1)	25 (8.9)	0.002
Chronic renal insufficiency^f^, *n* (%)	53 (8.9)	32 (9.8)	21 (7.5)	0.32
Dialysis dependence, *n* (%)	14 (2.3)	11 (3.4)	3 (1.1)	0.06
Autoimmune disease, *n* (%)	10 (1.7)	2 (0.6)	8 (2.9)	0.03
Cirrhosis, *n* (%)	26 (4.3)	14 (4.3)	12 (4.3)	0.99
Bacterial coinfection at admission, *n* (%)	205 (33.9)	118 (36.1)	87 (31.1)	0.19
Septic shock at admission^g^, *n* (%)	74 (12.3)	33 (10.1)	41 (14.6)	0.09
Pregnant or postpartum^h^, *n* (%)	30 (906)	24 (14.7)	6 (4.0)	0.001

*APACHE* Acute Physiology and Chronic Health Evaluation, *BMI* body mass index, *SOFA* Sequential Organ Failure Assessment, *COPD* chronic obstructive pulmonary disease, *day 1* first day in intensive care unit, *FiO*_*2*_ fraction of inspired oxygen, *PaO*_*2*_ partial pressure of oxygen in arterial blood;

^a^Mean (standard deviation) unless otherwise specified

^b^Denominators may vary for each category

^c^ *p* Value reflects comparison between patients treated and not treated with corticosteroids

^d^Obesity is defined as BMI >30 kg/m^2^

^e^Immune suppression; encompasses chemotherapy for malignancy, diagnosis of HIV/AIDS, and other immunosuppression

^f^Chronic renal insufficiency, defined as creatinine >1.5× normal

^g^Septic shock at admission as determined by the patient’s physician

^h^Female subset

**Table 2:** Cointerventions received according to corticosteroid treatment status among critically ill patients with H1N1pdm09

Cointervention	All patients^a^ (*n* = 607)	Not treated with corticosteroids (*n* = 327)	Treated with corticosteroids (*n* = 280)	*p* Value
Mechanical ventilation, day 1	392 (66.2)	212 (67.5)	180 (64.8)	0.48
Mechanical ventilation, any	535 (89.2)	275 (85.4)	260 (93.5)	0.001
Rescue oxygenation strategy, any	98 (16.1)	39 (11.9)	59 (21.1)	0.002
ECMO	18 (3.0)	10 (3.1)	8 (2.9)	0.88
HFOV	61 (10.1)	27 (8.3)	34 (12.1)	0.11
Nitric oxide	52 (8.6)	21 (6.4)	31 (11.1)	0.04
Prone ventilation	11 (1.8)	3 (0.9)	8 (2.9)	0.07
Antibiotic treatment, day 1	382 (63.9)	199 (60.9)	183 (65.4)	0.25
Antibiotic treatment, any	588 (96.9)	317 (96.9)	271 (96.8)	0.91
Neuraminidase inhibitor treatment, day 1	316 (52.1)	166 (50.8)	150 (53.6)	0.49
Neuraminidase inhibitor treatment, any	564 (92.9)	294 (89.9)	270 (96.4)	0.002
Vasopressor treatment, day 1	251 (41.4)	131 (40.1)	120 (42.9)	0.49
Vasopressor treatment, any	334 (55.0)	171 (52.3)	163 (58.2)	0.14

*ECMO* extracorporeal membrane oxygenation; *HFOV* high-frequency oscillation ventilation; *Day 1* first day in intensive care unit

Data are presented as number (%). Some patients received more than 1 rescue strategy.

^a^Denominators may vary for each category

Next, we repeated the multivariable logistic regression, including propensity to receive corticosteroids as a predictor variable for clinical outcomes. To further explore the association of corticosteroids on clinical outcomes, we performed an analysis of patients who received and did not received corticosteroids, with each steroid-receiving patient matched with a non–steroid-receiving patient, based on their propensity to receive corticosteroids. We used greedy matching without replacement algorithm with a caliper of 0.2 times the standard deviation of the logit-transformed propensity scores [38]. To test balance between the matched pairs based on the measured confounding variable, baseline differences were reexamined by way of matched pair analysis. Paired *t* and McNemar’s tests were used for the continuous and categorical variables, respectively, highlighting imbalance for only 1 of the 24 covariates (SOFA day 1), and this variable was subsequently removed. Characteristics of matched and unmatched patients were compared. Unadjusted survival curves were analyzed using the Kaplan-Meier method and compared using the log-rank test.

### Time-dependent variable adjustment using marginal structural models

Many patients are treated with steroids soon after ICU admission and data collection initiation, and do not allow investigation of the importance of time-dependent changes. Therefore, to perform time-dependent adjustments, we restricted analyses to patients who were alive at day 7 after ICU admission and had not yet received steroids. We used marginal structural models and inverse probability weighting to estimate the causal effect of steroids on mortality in the presence of time-dependent treatment and other potential confounders before steroid exposure [39, 40]. We therefore associated all potential predictor variables, including receipt of corticosteroids with a date (baseline variables on day 1 and subsequent measures of physiological state, occurrence of infection, receipt of ventilation, and hemodynamic or medication treatments on the day they were measured or administered) to establish a temporal relationship with death [39, 40]. Refining marginal structural models involves a two-step process to estimate weights. In step 1, we calculated stabilized weights using logistic regression models for the probability that each subject received his or her own treatment, being censored on day *k* of ICU stay, based on the previous treatment, baseline characteristics, and time-dependent covariates (Additional file 1: Appendix B). In the second step, we used a weighted logistic regression model (using the weights derived in step 1) (Additional file 2) to create generalized estimating equations to take into account the repeated measures nature of the data. We modeled the time-dependent intercept using restricted cubic splines with five knots for days since ICU admission [39, 40]. We modeled the probability of receiving steroid treatment with the assumption that once the patients were started on steroids they remained on the treatment (to approximate a randomized control trial where all events subsequent to receiving the allocated treatment are analyzed within that treatment group).

## Results

### Characteristics of patients receiving corticosteroids or not

From 756 total patients, 607 adults with H1N1pdm09-related critical illness from 47 of 51 participating Canadian hospitals met the eligibility criteria for this study (Fig. 1). Their mean age was 47.4 (standard deviation, 15.3) years, and 51.9 % were female. The mean number of comorbidities per patient was 3.2 (±2.3), with the most common being hypertension, pulmonary disease, diabetes, and obesity (Table 1). Mean APACHE II score was 20.6 (±10.0) and mean SOFA score was 11.4 (±3.7) at the onset of critical illness. Approximately 90 % of patients received mechanical ventilation, and 98 (16.1 %) patients received rescue oxygenation therapies: high-frequency oscillatory ventilation, inhaled nitric oxide, prone positioning, or extracorporeal membrane oxygenation (Table 2). Among all patients, 46.1 % were treated with corticosteroids, 92.9 % of patients received neuraminidase inhibitors, 96.9 % received antibiotics, and 55.0 % received vasopressors (Tables 2 and 3).

**Table 3:** Description of corticosteroid use among critically ill patients with H1N1pdm09

Medication variable	Median (q1, q3) or *n* (%)^a^
Corticosteroid treatment, *n* (%)	280 (46.1)
Corticosteroid prescribed, *n* (%)	
Prednisone	189 (34.4)
Methylprednisolone	177 (32.2)
Hydrocortisone	161 (29.3)
Dexamethasone	22 (4.0)
Cortisone	1 (0.2)
Duration of corticosteroids, days	7.0 (4.0, 13.0)
Dose, hydrocortisone equivalents per day^b^ (mg)	227 (154, 443)
Dose, hydrocortisone equivalents per day (mg/kg)	3.1 (1.7, 5.8)
Duration between onset of critical illness and corticosteroid initiation, days	0.0 (0.0, 3.0)
Duration between hospital admission and corticosteroid initiation, days	2.0 (1.0, 8.0)
Duration between onset of ventilation and corticosteroid initiation, days	1.0 (0.0, 3.0)
PaO_2_/F_i_O_2_ before corticosteroid administration^c^ (cmH_2_O)	140 (91, 220)
Positive End-Expiratory Pressure before corticosteroid administration^c^ (cmH_2_O)	10.0 (8.0, 14.0)
SOFA cardiovascular score before corticosteroid administration	2.0 (0.0, 3.0)

*SOFA* Sequential Organ Failure Assessment (SOFA score of 2 indicates patient receiving dopamine ≤5 μg/kg/minute or dobutamine any dose) [33], *FiO*_*2*_ fraction of inspired oxygen, *PaO*_*2*_ partial pressure of oxygen in arterial blood

^a^Denominators may vary for each category

^b^ http://www.medcalc.com/steroid.html

^c^PaO_2_/FiO_2_ ratio and positive end-expiratory pressure were recorded only on days 1, 3, 7, 14, and 28

There were substantial differences in baseline characteristics between patients who received corticosteroids and those who did not. Patients who received corticosteroids were older, had more comorbidities, and were more likely to have asthma, chronic obstructive pulmonary disease (COPD), and other pulmonary, immunosuppressive, or autoimmune conditions, but they were less likely to be pregnant (Table 1). Patients treated with corticosteroids were also more likely to receive mechanical ventilation, rescue oxygenation therapies, and neuraminidase inhibitors (Table 2).

### Corticosteroid use

The most commonly administered corticosteroids were prednisone, methylprednisolone, and hydrocortisone (Table 3). The median number of days of steroid of treatment was 7 (interquartile range 4, 13), and the median dose was 227 (154, 443) mg of hydrocortisone equivalents. Corticosteroids were started a median of 0 (0, 3) days from the onset of critical illness and 1 (0, 3) day from the initiation of mechanical ventilation. Median ratio of partial pressure of oxygen in arterial blood to fraction of inspired oxygen before corticosteroid administration was 140 (91, 220) cmH_2_O, and median positive end-expiratory pressure was 10 (8, 14) cmH_2_O. Independent factors associated with use of corticosteroids included preexisting pulmonary comorbidities, preexisting immunodeficiency, and septic shock at admission to ICU (Table 4). We did not find evidence of significant between-center differences.

**Table 4:** Predictors of corticosteroid administration among critically ill patients with H1N1pdm09

	Corticosteroid treatment			
Characteristics and cointerventions	Univariable analysis, OR (95 % CI)	*p* Value	Multivariable analysis, OR (95 % CI)	*p* Value
Age (per 1-year increase)	1.01 (1.00, 1.02)	0.04	1.06 (0.99, 1.02)	0.41
Female sex	1.18 (0.86, 1.63)	0.31	1.33 (0.92, 1.92)	0.13
APACHE II score (per 1-point increase)	1.01 (0.99, 1.03)	0.21	1.01 (0.99, 1.03)	0.37
Number of comorbidities per patient (per 1 increase)	1.17 (1.09, 1.26)	<0.001	1.04 (0.93, 1.15)	0.51
Pulmonary comorbidity	3.24 (2.27, 4.63)	<0.001	3.82 (2.48, 5.87)	<0.001
Cardiac disease	1.51 (0.96, 2.38)	0.07	1.00 (0.55, 1.81)	0.99
Immune suppression^a^	3.09 (1.46, 6.54)	0.003	3.58 (1.59, 8.06)	0.002
Septic shock at admission^b^	1.52 (0.93, 2.48)	0.09	2.20 (1.25, 3.88)	0.006
Bacterial coinfection at admission	0.80 (0.57, 1.13)	0.19	0.71 (0.48, 1.05)	0.09

*APACHE* Acute Physiology and Chronic Health Evaluation, *CI* confidence interval, *OR* odds ratio

^a^Immune suppression encompasses chemotherapy for malignancy, diagnosis of HIV/AIDS, and other immunosuppression

^b^Septic shock at admission, as determined by the patient’s physician

### Outcomes

In unadjusted analyses, patients receiving corticosteroids had higher hospital mortality and fewer ventilator-free and ICU-free days at 28 days, but no significant difference in nosocomial (bloodstream and respiratory) infections (Additional file 1: Appendix Table 5A). Both APACHE II score (OR 1.07, 95 % CI 1.04–1.10) and corticosteroid use (OR 1.85, 95 % CI 1.12–3.04) were associated with hospital mortality in multivariable logistic regression (Table 5) (*p* = 0.70, Hosmer-Lemeshow test). Adjusting for the likelihood of receiving corticosteroids by inclusion of a propensity score (Table 6 and Additional file 1: Appendix Tables 6A, 6B, 6C) yielded a similar association of corticosteroid treatment with hospital mortality (OR 1.71, 95 % CI 1.05–2.78) and ventilator-free and ICU-free days. However, after matching each patient who received corticosteroids with a patient of similar propensity score but who did not receive corticosteroids, there was no longer a significant association between corticosteroid treatment and hospital mortality (OR 1.52, 95 % CI 0.90–2.58) (Table 6). In addition, when we used a marginal structural model to examine the association of baseline and time-dependent variables over the first week of ICU admission until discharge from ICU on hospital mortality (Table 7; Additional file 1: Appendix B), only APACHE II score was an independent predictor of death; receipt of corticosteroids was not (OR 0.96, 95 % CI 0.28–3.28) (goodness-of-fit) (Additional file 1: Appendix B). In a sensitivity analysis using 4 days instead of 7, our findings were similar (OR 0.84, 95 % CI 0.26–2.64) (Additional file 1: Appendix Table 7A).

**Table 5:** Predictors of in-hospital mortality among critically ill patients with H1N1pdm09

	In-hospital mortality			
Clinical characteristics and cointerventions	Univariable analysis, OR (95 % CI)	*p* Value	Multivariable analysis, OR (95 % CI)	*p* Value
Age (1-year increase)	1.02 (1.01–1.04)	0.002	1.02 (1.00–1.03)	0.15
Female sex	0.94 (0.61–1.38)	0.77	0.92 (0.56–1.50)	0.73
Corticosteroid	1.82 (1.21–2.74)	0.004	1.85 (1.12–3.04)	0.02
APACHE II score (1-point increase)	1.07 (1.05–1.11)	<0.001	1.07 (1.04–1.10)	<0.001
PaO_2_/F_i_O_2_ ratio (cmH_2_O), day 1	1.00 (0.99–1.00)	0.004	0.99 (0.99–1.00)	0.06
Asthma	0.64 (0.37–1.11)	0.12	0.64 (0.33–1.26)	0.20
Autoimmune disease	3.21 (0.85–12.13)	0.09	3.02 (0.75–12.20)	0.12
Bacterial coinfection at admission	1.45 (0.96–2.20)	0.08	1.20 (0.72–1.98)	0.48

*APACHE* Acute Physiology and Chronic Health Evaluation; *Day 1* first day in intensive care unit, *OR* odds ratio, *CI* confidence interval, *FiO*_*2*_ fraction of inspired oxygen, *PaO*_*2*_ partial pressure of oxygen in arterial blood

**Table 6:** Outcomes of critically ill patients with H1N1pdm09 using various adjustment methodologies

Analysis	Odds ratio (95 % confidence interval)	*p* Value
Crude unadjusted analysis	1.82 (1.21–2.74)	0.004
Multivariate logistic regression analysis	1.85 (1.12–3.04)	0.02
Multivariate logistic regression analysis adjusted for propensity score to receive corticosteroids	1.71 (1.05–2.78)	0.03
Treatment groups matched on propensity to receive corticosteroids	1.52 (0.90–2.58)	0.12
Marginal structural model adjusting for baseline and time-dependent between-group differences over the first week of ICU admission until discharge or death	0.96 (0.28–3.28)	0.95

*ICU* intensive care unit

**Table 7:** Predictors of in-hospital mortality using adjustment for baseline and time-dependent between-group differences over the first week of ICU admission and until discharge from ICU among critically ill patients with H1N1pdm09

Variable	Rate ratio (95 % CI)	*p* Value
Corticosteroid use	0.96 (0.28–3.28)	0.95
APACHE II score (1-point increase)	1.07 (1.01–1.13)	0.02
SOFA score, day 1 (1-point increase)	0.97 (0.87–1.09)	0.63
Age (1-year increase)	1.00 (0.97–1.03)	0.87
Female sex	1.53 (0.65–3.83)	0.32
Asthma (yes vs no)	0.97 (0.27–3.52)	0.96
Autoimmune disease (yes vs no)	1.92 (0.54–6.81)	0.31

*APACHE* Acute Physiology and Chronic Health Evaluation, *SOFA* Sequential Organ Failure Assessment, *CI* confidence interval

In the final model (*n* = 286), we considered the following baseline variables upon examination of the predictors of outcome from other analyses (univariate, multivariate, and propensity matching) and accounting for overly correlated pairs of variables, including admission bacterial coinfection, SOFA score, APACHE II score, age, sex, asthma, autoimmune disease; and the following time-dependent variables SOFA (previous day), worsening ventilation (previous day or 2 prior days), positive blood or respiratory culture (previous day or 2 prior days), antibiotics started (previous day), and neuraminidase started (previous day).

## Discussion

To our knowledge, this is the largest observational study of H1N1pdm09-related critical illness to investigate the association of corticosteroid prescription on clinical outcomes. Using multiple methods to adjust for baseline differences between patients receiving and not receiving corticosteroids, we estimated a substantial and significant association between corticosteroids and increased mortality. However, adjusting for both baseline and time-dependent differences across the course of critical illness did not support an association between corticosteroid use and mortality.

Despite conflicting prior evidence of efficacy associated with corticosteroid use in critical illness, we found that nearly half of all patients with H1N1pdm09-related critical illness received this therapy. Most patients receiving corticosteroids in this study had moderate to severe ARDS. The frequency of prescription and median daily corticosteroid dose were similar to the experience reported in other series from Europe and Asia [20, 24]. We found that pulmonary disease, immune suppression, bacterial coinfection and septic shock at admission were independently associated with subsequent corticosteroid administration. These findings support the notion that COPD, asthma, and septic shock are other potential indications for corticosteroid use [41–44]. While there was substantial variability in prescribing practice at the patient level, we did not find substantial between-center differences, indicating that the majority of variability likely rests at the provider level.

Researchers in numerous observational studies have attempted to estimate the effect of corticosteroids upon clinical outcomes. In two recent methodologically rigorous studies, investigators similarly examined the association of corticosteroids and survival for patients with H1N1pdm09-related critical illness [20, 24]. These studies used a combination of multivariable logistic regression and propensity score matching to adjust for baseline differences, including severity of illness, between patients receiving and not receiving corticosteroids. One study considered steroids as a time-dependent (early vs late administration) variable to mitigate immortal time bias that arises from the requirement for patients to survive long enough to receive corticosteroids, possibly leading to an overestimation of a positive treatment effect. These studies also considered changes in severity of illness over the first 72 h of admission to the ICU (such that deteriorating patients may be more likely to be prescribed corticosteroids, possibly leading to an overestimation of an adverse treatment effect) [20]. However, a multitude of other factors that may change daily in critically ill patients (e.g., alveolar gas exchange, ventilator requirements, hemodynamics, infectious status, and initiation of other medications such as antibiotics or neuraminidase inhibitors) may also influence the decision to prescribe corticosteroids and were not accounted for in the previous studies.

We hypothesized that the association between corticosteroids and mortality in other studies might be due to the inability of multivariable analyses and propensity scores to adjust for unmeasured patient characteristics, confounding by indication, immortal time bias, and postbaseline time-dependent patient differences that influence the decision to prescribe corticosteroids [45]. Unobserved factors that affect assignment to treatment cannot be accounted for in matching procedures focused on variables at admission to ICU [45, 46]. Propensity matching ideally requires large samples with substantial overlap between treatment and control groups. Any hidden bias due to latent variables may remain after matching because the procedure controls only for observed variables and typically not for time-dependent between-group differences. Time-dependent differences are uncommonly accounted for but may have substantial influences on the subsequent decision to initiate treatment and on clinical outcomes. Marginal structural models estimate the causal effect of a time-dependent exposure on outcome in the presence of baseline covariates and time-dependent confounders and represent one method to account for time-dependent confounders [39, 40, 47]. When we applied such a model among a greater number of patients, using changes in such variables over the course of the first week in the ICU until discharge or death, we found no independent association of steroids with in-hospital mortality. These findings are in accordance with findings derived from some randomized controlled trials, such as a finding of no effect of early steroid use on mortality among patients with ARDS [18].

Our study has a number of strengths, including multicenter representation from all regions of Canada, a relatively large sample size, a priori definitions, data collection by trained research coordinators at each center, and consideration of baseline and time-dependent differences in the estimation of treatment effect. Furthermore, to address the possibility of unmeasured confounders, we used multivariable analyses employing propensity scores and case matching and adjustment for in-ICU, time-dependent, between-group differences. While we believe that these analytic approaches are incrementally valuable for estimating treatment effects from observational studies in a field where randomized trials are not yet forthcoming, it is unlikely that we have fully adjusted for all such differences.

Without a demonstrable benefit on survival, clinicians must consider other side effects of corticosteroids when contemplating corticosteroid prescription for patients with pandemic influenza. Multiple observational studies and controlled trials have demonstrated increased rates of neuromyopathy, impaired glycemic control, and the possibility of increased numbers of infections and/or the need for concomitant active infection surveillance, all of which may impair recovery from critical illness and are not well documented in most studies and trials of critically ill patients [15].

## Conclusions

Corticosteroids were commonly prescribed for H1N1pdm09-related critical illness. Adjusting for only baseline between-group differences suggested a significant increased risk of death associated with corticosteroids. However, after adjusting for time-dependent differences, we found no significant association between corticosteroids and mortality. Importantly, we have not found evidence to support the frequent use of corticosteroids among patients with H1N1pdm09-related critical illness in the absence of other evidence-based indications [15, 18]. Our findings provide more valid estimates of the influence of corticosteroids on outcomes for patients with influenza-related critical illness, add incremental information upon which clinicians can base clinical and research decision-making, and underscore the inherent challenges in estimating treatment effects on the basis of observational data.

## Key messages

- While observational studies have reported significant corticosteroid-associated mortality, corticosteroids have remained a common treatment in influenza-related critical illness.
- Among 607 patients with H1N1pdm09-related critical illness from 51 Canadian ICUs, corticosteroids were administered to 46.1 %.
- Compared with patients who did not receive corticosteroids, patients who received corticosteroids had higher hospital crude mortality; however, the odds ratio association between corticosteroid use and hospital mortality decreased from 1.85 with multivariate logistic regression, to 1.71 after adjustment for propensity score to receive corticosteroids, to 1.52 after case matching on propensity score, and to 0.96 using marginal structural modeling to adjust for time-dependent between-group differences.
- These findings highlight the challenges and importance in adjusting for baseline and time-dependent confounders when estimating clinical effects of treatments using observational studies.

## Additional files

Additional file 1: Appendix A (Table 5A. Unadjusted Clinical Outcomes among Critically Ill Patients with H1N1pdm09, Table 6A. Baseline Characteristics of Patients Matched by Propensity to Receive Corticosteroids among Critically Ill Patients with H1N1pdm09, Table 6B. Cointerventions Matched by Propensity to Receive Corticosteroids among Critically Ill Patients with H1N1pdm09, Table 6C. Outcome of Patients, Matched by Propensity to Receive Corticosteroids Among Critically Ill Patients with H1N1pdm09, Table 7A: Predictors of In-Hospital Mortality Using Adjustment for Baseline and Time-Dependent Between-Group Differences over the 4 Days of ICU Admission and Until Discharge From ICU Among Critically Ill Patients with H1N1pdm09) and Appendix B (Predictors of In-Hospital Mortality Among Critically Ill Patients with H1N1pdm09 Using Adjustment for Baseline and Time-Dependent Between-Group Differences) and Appendix C (Participating Hospitals) and Appendix D (Case Report Form). (ZIP 94 kb) Additional file 2: Figure S2. Marginal structural models box plot for the logarithm of the stabilized weights. Refining marginal structural models and evaluating goodness of fit involves a two-step process to estimate weights. First we calculated stabilized weights using logistic regression models for the probability that each subject received his or her own treatment, being censored on day *k* of ICU based on the previous treatment, baseline characteristics, and time-dependent covariates. Then the logarithms of stabilized weights that include information regarding steroid treatment as well as censoring are given. The distribution of the stabilized weights is centered at 1, and its variance increases over time, as expected. (DOCX 33 kb)

## Abbreviations

APACHE: Acute Physiology and Chronic Health Evaluation
ARDS: acute respiratory distress syndrome
BMI: body mass index
CI: confidence interval
COPD: chronic obstructive pulmonary disease
ECMO: extracorporeal membrane oxygenation
FiO_2_: fraction of inspired oxygen
HFOV: high-frequency oscillation ventilation
H1N1pdm09: 2009 pandemic influenza A(H1N1)
ICU: intensive care unit
OR: odds ratio
PaO_2_: partial pressure of oxygen in arterial blood
SOFA: Sequential Organ Failure Assessment
