# Supplementary material for: The influence of corticosteroid treatment on the outcome of influenza A(H1N1pdm09)-related critical illness
Source: Crit Care. 2016 Mar 30;20:75. doi: 10.1186/s13054-016-1230-8 (PMC4818504; doi:10.1186/s13054-016-1230-8)
Supplement: Additional file 1: — Appendix A (Table 5A. Unadjusted Clinical Outcomes among Critically Ill Patients with H1N1pdm09, Table 6A. Baseline Characteristics of Patients Matched by Propensity to Receive Corticosteroids among Critically Ill Patients with H1N1pdm09, Table 6B. Cointerventions Matched by Propensity to Receive Corticosteroids among Critically Ill Patients with H1N1pdm09, Table 6C. Outcome of Patients, Matched by Propensity to Receive Corticosteroids Among Critically Ill Patients with H1N1pdm09, Table 7A: Predictors of In-Hospital Mortality Using Adjustment for Baseline and Time-Dependent Between-Group Differences over the 4 Days of ICU Admission and Until Discharge From ICU Among Critically Ill Patients with H1N1pdm09) and Appendix B (Predictors of In-Hospital Mortality Among Critically Ill Patients with H1N1pdm09 Using Adjustment for Baseline and Time-Dependent Between-Group Differences) and Appendix C (Participating Hospitals) and Appendix D (Case Report Form). (ZIP 94 kb) [file 13054_2016_1230_MOESM1_ESM.zip › Additional File (Appendix A, B, C).docx]

**Appendix A. Tables.**

**Table 5A Unadjusted Clinical Outcomes Among Critically Ill Patients with H1N1pdm09**

| **Baseline characteristics^*^** | **Overall^**^**  **N=604** | **Not Treated with**  **Corticosteroids**  **N=324** | **Treated**  **with**  **Corticosteroids**  **N=280** | **P-value** |
| --- | --- | --- | --- | --- |
| Hospital Mortality, N (%) | 121 (20.6) | 51 (16.4) | 70 (25.5) | 0.007 |
| Ventilator-Free Days at 28 Days | 14.2 (10.5) | 15.7 (10.1) | 12.5 (10.7) | <0.001 |
| ICU-Free Days at 28 Days | 11.5 (9.8) | 13.2 (9.4) | 9.5 (9.9) | <0.001 |
| Positive Respiratory or  Bloodstream Cultures, N (%) | 181 (31.5) | 89 (28.7) | 92 (34.7) | 0.12 |

^*^Mean (SD) unless specified;^**^Denominators may vary for each category;

**Table 6A Baseline Characteristics of Patients Matched by Propensity to Receive Corticosteroids Among Critically Ill Patients with H1N1pdm09**

| **Baseline characteristics, mean (SD)^*^** | **All**  **Patients^**^**  **N=332** | **Not Treated with**  **Corticosteroids**  **N=166** | **Treated**  **with**  **Corticosteroids**  **N=166** | **P-value** |
| --- | --- | --- | --- | --- |
| Age | 46.7 (14.6) | 46.6 (14.8) | 46.6 (14.4) | 0.88 |
| Female Sex, N (%) | 167 (50.3) | 85 (51.2) | 82 (49.4) | 0.74 |
| BMI | 32.0 (10.2) | 31.7 (10.2) | 32.4 (10.3) | 0.74 |
| APACHE II score | 21.0 (10.1) | 20.8 (9.7) | 21.2 (10.5) | 0.65 |
| SOFA Score, Day 1 | 11.4 (3.7) | 11.2 (3.7) | 11.6 (3.7) | 0.07 |
| SOFA Cardiovascular Score, Day 1, n=317 | 1.6 (1.5) | 1.6 (1.4) | 1.6 (1.5) | 0.94 |
| PaO_2_ / FiO_2_ ratio, Day 1 | 151 (94) | 154 (96) | 148 (91) | 0.72 |
| Tidal Volume (ml), Day 1 | 518 (135) | 514 (116) | 522 (152) | 0.66 |
| Tidal Volume per predicted body weight (ml/kg), Day 1 | 6.2 (2.0) | 6.2 (2.2) | 6.1 (1.9) | 0.75 |
| Positive End Expiratory Pressure, (cm H2O), Day 1 | 11.1 (4.7) | 10.9 (4.5) | 11.3 (4.9) | 0.53 |
| Patients with Comorbidities, (Any), N (%) | 299 (90.1) | 148 (89.2) | 151 (91.0) | 0.58 |
| Number of comorbidities per patient, N (%) | 3.2 (2.3) | 3.2 (2.3) | 3.2 (2.4) | 0.71 |
| Asthma, N (%) | 69 (20.8) | 35 (21.8) | 34 (20.5) | 0.89 |
| COPD, N (%) | 44 (13.3) | 22 (13.3) | 22 (13.3) | 1.00 |
| Pulmonary, N (%) | 103 (31.0) | 53 (31.9) | 50 (30.1) | 0.66 |
| Cardiac Disease, N (%) | 47 (14.2) | 25 (15.1) | 22 (13.3) | 0.62 |
| Hypertension, N (%) | 100 (30.1) | 48(28.9) | 52 (31.3) | 0.63 |
| Obesity^a^, N (%) | 82 (24.7) | 38 (22.9) | 44 (26.5) | 0.41 |
| Diabetes, N (%) | 87 (26.2) | 44 (26.5) | 43 (25.9) | 0.91 |
| Immune Suppression^b^,  N(%) | 21 (6.3) | 10 (6.0) | 11 (6.6) | 0.82 |
| Chronic Renal  Insufficiency^c^, N (%) | 23 (6.9) | 11 (6.6) | 12 (7.2) | 0.83 |
| Dialysis Dependence, N (%) | 5 (1.5) | 3 (1.8) | 2 (1.2) | 0.66 |
| Autoimmune Disease, N (%) | 2 (0.6) | 2 (1.2) | 0 | - |
| Cirrhosis, N (%) | 20 (6.0) | 9 (5.4) | 11 (6.6) | 0.66 |
| Bacterial co-infection at  admission, N (%) | 115 (34.6) | 61 (36.8) | 54 (32.5) | 0.68 |
| Septic Shock at Admission^d^, N (%) | 53 (16.0) | 24 (14.5) | 29 (17.5) | 0.44 |
| Pregnancy or Post-Partum^e^, N (%) | 18 (10.8) | 12 (14.1) | 6 (7.2) | 0.74 |

^*^Mean (SD) unless specified;^**^Denominators may vary for each category; ^a^Obesity = BMI > 30 kg/m^2^; ^b^Immune suppression - encompasses chemotherapy for malignancy, diagnosis of HIV/AID and other immunosuppression; ^c^Chronic renal insufficiency - defined as creatinine >1.5 x normal; ^d^Septic shock at admission - as determined by the patients physician;^e^Female subset.

Definitions: BMI - Body Mass Index; APACHE - acute physiology and chronic health assessment; SOFA - Sequential Organ Failure Assessment; COPD - Chronic Obstructive Pulmonary Disease; Day 1 – first day in ICU

**Table 6B Co-Interventions Matched by Propensity to Receive Corticosteroids Among Critically Ill Patients with H1N1pdm09**

| **Co-Intervention, N (%)** | **All**  **Patients^*^**  **N=332** | **Not Treated with**  **Corticosteroids**  **N=166** | **Treated with Corticosteroids**  **N=166** | **P-value** |
| --- | --- | --- | --- | --- |
| Mechanical Ventilation, Day 1 | 231 (69.6) | 117 (70.5) | 114 (68.7) | 0.71 |
| Rescue Ventilation Strategy, Any | 68 (20.5) | 33 (19.9) | 35 (21.1) | 0.78 |
| ECMO | 12 (3.6) | 7 (4.2) | 5 (3.0) | 0.56 |
| HFO | 43 (13.0) | 22 (13.3) | 21 (12.7) | 0.87 |
| Nitric Oxide | 38 (11.5) | 19 (11.5) | 19 (11.5) | 1.00 |
| Prone ventilation | 10 (3.0) | 3 (1.8) | 7 (4.2) | 0.21 |
| Antibiotic Treatment, Day 1 | 214 (64.5) | 105 (63.3) | 109 (65.7) | 0.63 |
| Neuraminidase Inhibitor Treatment, Day 1 | 187 (56.3) | 93 (56.0) | 94 (56.6) | 0.91 |
| Vasopressor treatment, Day 1 | 144 (43.4) | 69 (41.6) | 75 (45.2) | 0.31 |

**^*^**Denominators may vary for each category; Definitions: ECMO - Extra-Corporeal Membrane Oxygenation; HFO - High Frequency Oscillation; Day 1 – first day in ICU

**Table 6C** **Outcome of Patients, Matched by Propensity to Receive Corticosteroids Among Critically Ill Patients with H1N1pdm09**

| **Clinical Outcome^*^** | **Overall^**^**  **N=332** | **Not Treated with Corticosteroids**  **N=166** | **Treated with Corticosteroids**  **N=166** | **P-value** |
| --- | --- | --- | --- | --- |
| Hospital Mortality, N (%), | 68 (20.8) | 27 (16.7) | 41 (24.9) | 0.12 |
| Ventilator-Free Days at 28 Days | 13.4 (10.3) | 14.6 (9.9) | 12.2 (10.7) | 0.03 |
| ICU-Free Days at 28 Days | 10.6 (9.6) | 12.7 (9.1) | 8.9 (9.7) | 0.002 |
| Hospital-acquired Positive Respiratory or Bloodstream Cultures, N (%), n=315 | 105 (33.3) | 49 (31.0) | 56 (35.7) | 0.50 |

^*^Mean (SD) unless specified;^**^Denominators may vary for each category

**Table 7A Predictors of In-Hospital Mortality Using Adjustment for Baseline *and* Time-Dependent Between-Group Differences over the 4 Days of ICU Admission and Until Discharge From ICU Among Critically Ill Patients with H1N1pdm09***

| **Variable** | **Rate Ratio (95% CI)** | **P-value** |
| --- | --- | --- |
| Corticosteroid Use | 0.84 (0.26, 2.64) | 0.76 |
| APACHE II Score (1 point increase) | 1.04 (0.98, 1.11) | 0.17 |
| SOFA score, Day 1 (1 point increase) | 1.00 (0.89, 1.13) | 0.99 |
| Age (1 year increase) | 1.01 (0.98, 1.03) | 0.69 |
| Female Sex | 1.49 (0.64, 3.47) | 0.36 |
| Asthma (Yes vs. No) | 0.84 (0.23, 3.08) | 0.79 |
| Autoimmune Disease (Yes vs. No) | 1.98 (0.56, 7.07) | 0.29 |

Definitions: APACHE - acute physiology and chronic health assessment; SOFA - Sequential Organ Failure Assessment

* The final model (N=297) considered the following baseline variables upon examination of the predictors of outcome from other analyses (univariate, multivariate, and propensity matching) and accounting for overly correlated pairs of variables, including: admission bacterial co-infection, SOFA score, APACHE II score, age, sex, asthma, autoimmune disease; and, the following time-dependent variables, SOFA (previous day), worsening ventilation (previous day or 2 prior days), positive blood or respiratory culture (previous day or 2 prior days), antibiotics started (previous day), neuraminidase started (previous day).

**Appendix B Predictors of In-Hospital Mortality Among Critically Ill Patients with H1N1pdm09 Using Adjustment for Baseline *and* Time-Dependent Between-Group Differences***

Steroid exposure was modeled using marginal structural models to examine independent associations between baseline and time-dependent variables on in-hospital mortality.

**Assumptions and Procedures**

**SOFA**

1. Impute the SOFA respiratory score based on a clinical decision if one of PaO_2_ or FiO_2_ is missing.
2. If respiratory score available even if not all the other scores were available they were considered as being zero and SOFA was calculated based on the available scores.
3. Day 2 SOFA not available for wave 1 patients (spring 2009) so if either day 1 or day 3 was available, these were used.
4. If any of the SOFA day 1-3 were missing we look to the closed value from the other days 1-3.
5. If the SOFA is missing at the end of ICU follow-up (e.g. If length of stay in ICU=29 days and day 28 SOFA is missing then use the latest sofa available day 14 or day 7 or day 3).
6. If the data is missing in between 2 closely aligned days (e.g. between day 3 and 7) use linear interpolation to calculate SOFA.

**Ventilation**

1. Worsening of ventilation – we consider the worsening on the day, previous day and 2 days before
   1. For the first 2 days – worsening in the previous day was compared to day 0.
   2. If rescue oxygenation or ventilation therapies were used (e.g. ECMO/HFO/prone positioning/inhaled nitric oxide, etc.) on the first ICU day we assumed worsening from day 0 as we do not have information prior to ICU admission, and in most all cases, these therapies would be used in the ICU after admission but not beforehand.

**Variables in the model**

**Baseline Covariates.** Chosen upon examination of the predictors of outcome from other analysis (univariate, multivariate, propensity matching) and accounting for overly correlated pairs of variables.

1. Bacterial Co-infection at baseline
2. SOFA at baseline
3. APACHE at baseline
4. Age
5. Gender
6. Asthma
7. Autoimmune disease

Time- dependent variables:

1. SOFA previous day
2. Worsening ventilation previous day
3. Worsening ventilation 2 days before
4. Previous day blood or respiratory positive culture
5. 2 days prior blood or respiratory positive culture
6. Previous day antibiotics started
7. Previous day neuraminidase started

**Appendix C. Participating Hospitals**

1. Bonavista Community Health
2. CHA – Hôpital de l’Enfant-Jesus
3. Carbonear General Hospital
4. Centre HospitalierUniversaire de Sherbrooke(CHUS)
5. Grace Hospital
6. Grey Nuns Hospital
7. Hamilton Health Sciences Centre1
8. Hamilton St Joseph's Hospital
9. Health Sciences Centre- Critical Care -Newfoundland
10. Hopital du Sacre-Coeur de Montreal
11. Hospital Maisoneuve-Rosemont, Montreal
12. Hospital Regional de Sudbury Regional Hospital
13. Hospital for Sick Children
14. Hôpital Charles LeMoyne
15. InstitutUniversitaire de cardiologie et de pneumologie de Québec
16. Jewish General Hospital
17. Kingston General Hospital1
18. McMaster University Medical Centre (MUMC)
19. Montreal General Hospital
20. Mount Sinai Hospital
21. North Bay General Hospital
22. North York General Hospital
23. Ottawa Hospital Research Institute - Ottawa General
24. Ottawa Hospital- Centre for Transfusion and Critical Care Research (Civic Campus)
25. Queen Elizabeth II Health Sciences Centre
26. Royal Alexandra Hospital
27. Royal Columbian Hospital
28. Royal Jubilee Hospital
29. Royal University Hospital
30. Royal Victoria Hospital
31. Sault Area Hospital
32. St Clare's Mercy Hospital
33. St Joseph's Health Centre
34. St Joseph's Healthcare
35. St. Boniface General Hospital
36. St. Michael’s Hospital- Critical Care Medicine
37. St. Paul's Hospital - Saskatoon
38. St. Paul's Hospital- Center for Health Evaluation and Outcome Sciences
39. Stanton Territorial Health Authority
40. Sunnybrook Hospital- Depts of Medicine and Critical Care Medicine
41. Surrey Memorial Hospital
42. The Montreal Children's Hospital
43. Thunder Bay Regional Health Sciences Centre
44. University Health Network - Toronto General Hospital
45. University Health Network- Toronto Western Hospital- Critical Care Medicine
46. University of Alberta Hospital
47. University of Calgary-Foothills Medical Centre Room 1105
48. Vancouver General Hospital- Critical Care Medicine
49. Victoria General Hospital
50. Whitehorse General Hospital
51. Winnipeg Health Sciences Centre- Section of Critical Care Medicine- GE706
