# Supplementary material for: The influence of corticosteroid treatment on the outcome of influenza A(H1N1pdm09)-related critical illness
Source: Crit Care. 2016 Mar 30;20:75. doi: 10.1186/s13054-016-1230-8 (PMC4818504; doi:10.1186/s13054-016-1230-8)
Supplement: Additional file 1: — Appendix A (Table 5A. Unadjusted Clinical Outcomes among Critically Ill Patients with H1N1pdm09, Table 6A. Baseline Characteristics of Patients Matched by Propensity to Receive Corticosteroids among Critically Ill Patients with H1N1pdm09, Table 6B. Cointerventions Matched by Propensity to Receive Corticosteroids among Critically Ill Patients with H1N1pdm09, Table 6C. Outcome of Patients, Matched by Propensity to Receive Corticosteroids Among Critically Ill Patients with H1N1pdm09, Table 7A: Predictors of In-Hospital Mortality Using Adjustment for Baseline and Time-Dependent Between-Group Differences over the 4 Days of ICU Admission and Until Discharge From ICU Among Critically Ill Patients with H1N1pdm09) and Appendix B (Predictors of In-Hospital Mortality Among Critically Ill Patients with H1N1pdm09 Using Adjustment for Baseline and Time-Dependent Between-Group Differences) and Appendix C (Participating Hospitals) and Appendix D (Case Report Form). (ZIP 94 kb) [file 13054_2016_1230_MOESM1_ESM.zip › Additional Files (Appendix D).docx]

**Appendix D. Case Report Form**

**ICU Flu Study - Case Report Form**

Eligibility Criteria – To be eligible, a patient should fulfill at least one criterion from both (A) AND (B) – please specify the criteria in the check boxes.

**A. Has Influenza**

□  **Confirmed Influenza B**- any specimen yielding influenza B by PCR and/or viral culture.

□  **Confirmed Influenza A (not sub-typed)** - any specimen yielding influenza A by PCR and/or viral culture, where sub-typing is not possible or available.

□  **Confirmed A(H1N1)v** –any specimen yielding A(H1N1)v by PCR and/or viral culture (EIA/DFA may or may not be positive).

□  **Confirmed Influenza A NOT (H1N1)v** (specify other subtype if known: ______________) –any specimen yielding  influenza  A(H3N2 or seasonal H1N1) by PCR and/or viral culture (EIA/DFA may or may not be positive).

□  **Probable Influenza A**–any specimen yielding influenza A by EIA/ELISA/DFA, where viral culture and PCR were not performed (Note: if viral culture or PCR is performed and positive, case is confirmed. If PCR and culture for influenza are negative, then the case is not an influenza case - the rapid test is assumed to be a false positive).

□  **Probable Influenza B**–any specimen yielding influenza B by EIA/ELISA/DFA, where viral culture and PCR were not performed (Note: if viral culture or PCR is performed and positive, case is confirmed. If PCR and culture for influenza are negative, then the case is not an influenza case - the rapid test is assumed to be a false positive).

□  **Possible/Suspected influenza A(H1N1)v** –when influenza A(H1N1)v is not detected but which occurs in a household or other close contact of a patient with laboratory confirmed A(H1N1v), or other reason to suspect A(H1N1)v, with symptom onset within 4 days of last exposure. (Note: where possible, in this circumstance, acute and convalescent serology will be obtained; if acute and convalescent serology are obtained, the case classification will be changed to confirmed if serology indicates a 4 fold increase in titer, and to not influenza if seroconversion does not occur).

**B. Is Critically Ill**

**□** Has been admitted to an Adult or Pediatric intensive care unit (ICU) / area of the hospital where critically ill patients receive treatment OR

□ Has received invasive or non-invasive mechanical ventilation OR

□ Has received continuous intravenous vasoactive medications OR

□ Has another feature that you believe classified them as critically ill in your setting. Specify: ______________________________________________________________________________.

**ICU Flu Study - Case Report Form**

**Demographic details**

| Sex: | Male Female |
| --- | --- |
| Age *or* Year of Birth: | ______ years or months or weeks  or year of birth ______(yyyy) |
| Health Care Worker: | Yes No Unknown |
| Body Weight (measure or estimate): | _____ kg OR _____ lbs |
| Height (measure or estimate): | _____ cm OR _____ inches |
| Influenza Vaccination History (check if yes): | 2008/2009 2007/2008 2006/2007 |
| Received Influenza H1N1 vaccine? | Yes No What month _____ Uncertain |
| Nosocomial acquisition | Yes No |
| Ethnicity/Race | White/Caucasian  Black (e.g. African, Haitian, Jamaican, Somali, etc)  □ Asian (e.g. Chinese, Japanese, Vietnamese, Cambodian, Indonesian, Laotian, Korean, Filipino, etc)  South Asian (e.g. Indian, Pakistani, Sri Lankan, Bangladeshi, etc)  Arab/West Asian (e.g. Armenian, Egyptian, Iranian, Lebanese, Moroccan, etc)  □ Latin American (e.g. Mexican, Central/South American, etc)  North American Indian □ Metis □ Inuit  Australian/New Zealand Aboriginal  Unknown / Mixed Other: ________________ |
| Source of Admission | ER Ward Step-up/down Unit Post-op Unit  OR – Scheduled OR – Unscheduled  Other Hospital ER/Ward Other Hospital ICU |

**Symptoms At Time-of-Presentation to Current Hospital Admission**

| Fever (Example ≥ 38^o^C or ≥ 100.4 ^o^F) | Yes No |
| --- | --- |
| Nausea | Yes No |
| Vomiting | Yes No |
| Diarrhea | Yes No |
| Headache | Yes No |
| Myalgias | Yes No |
| Weakness | Yes No |
| Wheeze | Yes No |
| Cough | Yes No |
| Shortness of breath | Yes No |
| Purulent sputum | Yes No |
| Hemoptysis or blood in sputum | Yes No |

**ICU Flu Study - Case Report Form**

**Current Co-morbidities/Conditions**

| Angina Yes No | Gastrointestinal Disease Yes No |
| --- | --- |
| Myocardial Infarction Yes No | Cancer, non-metastatic Yes No |
| Congestive Heart Failure - Yes No | Cancer, metastatic Yes No |
| Congestive Heart Failure – severe Yes No | Leukemia Yes No |
| Arrhythmia Yes No | Lymphoma Yes No |
| Valvular Heart Disease Yes No | AIDS Yes No |
| Congenital Heart Disease Yes No | Organ Transplantation Yes No |
| Hypertension Yes No | Corticosteroid use Yes No |
| Peripheral Vascular Disease Yes No | Chemotherapy Yes No |
| Cerebrovascular Disease Yes No | Other Immunosuppression Yes No |
| Diabetes (I/II, no end organ damage) Yes No | Specify: |
| Diabetes (I/II, end organ damage) Yes No | Systemic Autoimmune Disease Yes No |
| Renal Insufficiency (Cr > 1.5 x normal) Yes No | Rheumatologic Disease Yes No |
| Dialysis Dependency Yes No | Spina Bifida Yes No |
| Obesity (BMI >30kg/m^2^) Yes No | Dementia Yes No |
| Hyperlipidemia Yes No | Seizure Disorder Yes No |
| COPD Yes No | Paralysis Yes No |
| Asthma Yes No | Scoliosis Yes No |
| Bronchopulmonary Dysplasia Yes No | Cerebral Palsy/Development Delay Yes No |
| Other Chronic Lung Disease Yes No | Alcohol abuse Yes No |
| Chronic Lung Disease (any) – severe Yes No | IV drug abuse Yes No |
| Liver Disease - No portal HTN Yes No | Smoker (ever) Yes No |
| Liver Disease - Portal HTN/cirrhosis Yes No | Other: __________________________________ |

**Co-Presenting Illness (i.e. Because of, in addition to, *or* instead of influenza):**

| Pneumonia (non-influenza) Yes No | COPD Yes No |
| --- | --- |
| Bloodstream infection Yes No | Asthma Yes No |
| Urinary Infection Yes No | Renal Insufficiency (Cr 1.5x normal) Yes No |
| Congestive Heart Failure Yes No | Fluid/Electrolyte Disorder Yes No |
| Myocardial Infarction / Angina Yes No | Rhabdomyolysis Yes No |
| Arrhythmia Yes No | Gastrointestinal Bleeding Yes No |
| Peripheral Vascular Disease Yes No | Severe Pancreatitis Yes No |
| Cerebrovascular Disease Yes No | Septic Shock Yes No |
| Hepatic Disease Yes No | Surgery Yes No |
| Altered level of consciousness Yes No | Site of Surgery: |
| Diabetes (Type I or II) Complication Yes No | Other: |

**ICU Flu Study - Case Report Form**

**Treatments (at any time during/immediately preceding Influenza illness)**

| **Oseltamivir** course # 1 | Yes No | Start: **____/____/_____** Stop: **____/____/_____** (dd/mm/yyyy) | _____ mg OD BID |
| --- | --- | --- | --- |
| course # 2 | Yes No | Start: **____/____/_____** Stop: **____/____/_____** (dd/mm/yyyy) | _____ mg OD BID |
| course # 3 | Yes No | Start: **____/____/_____** Stop: **____/____/_____** (dd/mm/yyyy) | _____ mg OD BID |
| course # 4 | Yes No | Start: **____/____/_____** Stop: **____/____/_____** (dd/mm/yyyy) | _____ mg OD BID |
| **Zanamivir** course # 1 | Yes No | Start: **____/____/_____** Stop: **____/____/_____** (dd/mm/yyyy) | _____ mg OD BID |
| course # 2 | Yes No | Start: **____/____/_____** Stop: **____/____/_____** (dd/mm/yyyy) | _____ mg OD BID |
| course # 3 | Yes No | Start: **____/____/_____** Stop: **____/____/_____** (dd/mm/yyyy) | _____ mg OD BID |
| course # 4 | Yes No | Start: **____/____/_____** Stop: **____/____/_____** (dd/mm/yyyy) | _____ mg OD BID |
| **Amantadine** | Yes No | Start: **____/____/_____** Stop: **____/____/_____** (dd/mm/yyyy) | Dose: |
| **Rimantadine** | Yes No | Start: **____/____/_____** Stop: **____/____/_____** (dd/mm/yyyy) | Dose: |
| **Other** Influenza-specific Rx 1 | Yes No | **____/____/_____** (dd/mm/yyyy) | Name:  Dose: |
| **Other** Influenza-specific Rx 2 | Yes No | **____/____/_____** (dd/mm/yyyy) | Name:  Dose: |
| **Corticosteroid** course #1 | Yes No | Start: **____/____/_____** Stop: **____/____/_____** (dd/mm/yyyy) | Name:  Dose: |
| course # 2 | Yes No | Start: **____/____/_____** Stop: **____/____/_____** (dd/mm/yyyy) | Name:  Dose: |
| **Neuromuscular blockade**  course # 1 | Yes No | Start: **____/____/_____** Stop: **____/____/_____** (dd/mm/yyyy) |  |
| course # 2 | Yes No | Start: **____/____/_____** Stop: **____/____/_____** (dd/mm/yyyy) | Name:  Dose: |
| **Antibiotic** # 1 | Yes No | Start: **____/____/_____** Stop: **____/____/_____** (dd/mm/yyyy) | Name:  Dose: |
| **Antibiotic** # 2 | Yes No | Start: **____/____/_____** Stop: **____/____/_____** (dd/mm/yyyy) | Name:  Dose: |
| **Antibiotic** # 3 | Yes No | Start: **____/____/_____** Stop: **____/____/_____** (dd/mm/yyyy) | Name:  Dose: |
| **Antibiotic** # 4 | Yes No | Start: **____/____/_____** Stop: **____/____/_____** (dd/mm/yyyy) | Name:  Dose: |
| **Antibiotic** # 5 | Yes No | Start: **____/____/_____** Stop: **____/____/_____** (dd/mm/yyyy) | Name:  Dose: |
| **Antibiotic** # 6 | Yes No | Start: **____/____/_____** Stop: **____/____/_____** (dd/mm/yyyy) | Name:  Dose: |
| **Antibiotic** #7 | Yes No | Start: **____/____/_____** Stop: **____/____/_____** (dd/mm/yyyy) | Name:  Dose: |
| **Antibiotic** #8 | Yes No | Start: **____/____/_____** Stop: **____/____/_____** (dd/mm/yyyy) | Name:  Dose: |
| **Antibiotic** #9 | Yes No | Start: **____/____/_____** Stop: **____/____/_____** (dd/mm/yyyy) | Name:  Dose: |
| **Antibiotic** # 10 | Yes No | Start: **____/____/_____** Stop: **____/____/_____** (dd/mm/yyyy) | Name:  Dose: |

**ICU Flu Study - Case Report Form**

**Time Course and Outcomes**

| Date of onset of **initial symptoms** | ____/____/____ (dd/mm/yyyy) |
| --- | --- |
| **Hospital admission** date | ____/____/____ (dd/mm/yyyy) |
| **ICU admission** date | ____/____/____ (dd/mm/yyyy) |
| **Invasive mechanical ventilation**? | Yes No |
| **Non-invasive mechanical ventilation**? | Yes No |
| **Date any ventilation** initiated | ____/____/____ (dd/mm/yyyy) |
| **Date of initial separation from any ventilation** | ____/____/____ (dd/mm/yyyy) |
| **Was patient Re-intubated/ventilated/?** | Yes No |
| If yes, date of **final separation** from ventilation? | ____/____/____ (dd/mm/yyyy) |
| Was patient treated with **HFO?** | Yes No |
| Start Date | ____/____/____ (dd/mm/yyyy) |
| Stop Date | ____/____/____ (dd/mm/yyyy) |
| Was patient treated with **ECMO?** | Yes No |
| Start Date | ____/____/____ (dd/mm/yyyy) |
| Stop Date | ____/____/____ (dd/mm/yyyy) |
| Was patient treated with **Nitric Oxide?** | Yes No |
| Start Date | ____/____/____ (dd/mm/yyyy) |
| Stop Date | ____/____/____ (dd/mm/yyyy) |
| Was patient treated with **Prone Ventilation**? | Yes No |
| Start Date | ____/____/____ (dd/mm/yyyy) |
| Stop Date | ____/____/____ (dd/mm/yyyy) |
| **Date of initial ICU discharge** (if applicable) | ____/____/____ (dd/mm/yyyy) |
| **Was patient re-admitted to ICU?** | Yes No |
| If yes, date of **final discharge from ICU**? | ____/____/____ (dd/mm/yyyy) |
| **Alive at day 90?** | Yes No |
| Requires Supplemental O_2_? | Yes No |
| Requires Ventilation? | Invasive Yes No Non-Invasive Yes No |
| **Date of live hospital discharge (if applicable)** | ____/____/____ (dd/mm/yyyy) |
| Discharged to: | Home Chronic Care Facility |
|  | Acute Care Hospital Rehabilitation Facility |
| **Tracheostomy during hospitalization?** | Yes No ____/____/____ (dd/mm/yyyy) |
| **If Patient Died, Please Complete the Following** |  |
| Date of Death | ____/____/____ (dd/mm/yyyy) |

**Pregnancy During Illness**

| **Is Patient Currently Pregnant?** | Yes No Unknown |
| --- | --- |
| **Patient Post-Partum (within 42 days)?** | Yes No |
| Delivery Mode | Not delivered C-Section Spont. Vaginal  Assisted Vaginal Breech Vaginal |
| Was Labour Induced by Healthcare Team | Yes No |
| Gestational Age At Delivery | _______weeks |
| Fetal Outcome | Live birth Neonatal ICU admission  Neonatal death Stillbirth  Spontaneous abortion Therapeutic abortion |
| Congenital malformations | Yes No |
| Birth weight (grams) |  |
| APGAR score ( /10) (best) |  |

**ICU Flu Study - Case Report Form**

| **Primary Cause of Death (check ONE)?** | **Contributory Cause(s) of Death (check ANY)?** |
| --- | --- |
| Yes No Acute Lung Injury | Yes No Acute Lung Injury |
| Yes No Multi-organ Dysfunction Syndrome | Yes No Multi-organ Dysfunction Syndrome |
| Yes No Pneumonia | Yes No Pneumonia |
| Yes No Myocardial Infarction | Yes No Myocardial Infarction |
| Yes No Congestive Heart Failure | Yes No Congestive Heart Failure |
| Yes No Dysrhythmia | Yes No Dysrhythmia |
| Yes No Chronic Obstructive Lung Disease | Yes No Chronic Obstructive Lung Disease |
| Yes No Pulmonary Emboli | Yes No Pulmonary Emboli |
| Yes No Cerebrovascular Disease | Yes No Cerebrovascular Disease |
| Yes No Renal Failure | Yes No Renal Failure |
| Yes No Liver Failure | Yes No Liver Failure |
| Yes No Malignancy | Yes No Malignancy |
| Yes No Septic Shock | Yes No Septic Shock |
| Yes No Myocarditis | Yes No Myocarditis |
| Yes No Myocardial Infarction | Yes No Myocardial Infarction |
| Yes No Cardiogenic Shock | Yes No Cardiogenic Shock |
| Yes No Cerebral edema | Yes No Cerebral edema |
| Other (specify): | Other (specify): |

Microbiology (document organism only if a) not a probable contaminant/colonizer and b) associated with compatible clinical syndrome (e.g. septic shock, suspected line infection, UTI, bacterial pneumonia, etc)

| **Positive *blood* culture** (specify organism *Genus* *species*) at any point during hospitalization |
| --- |
| 1. ____/____/____(dd/mm/yyyy) 6. ____/____/____(dd/mm/yyyy) |
| 2. ____/____/____(dd/mm/yyyy) 7. ____/____/____(dd/mm/yyyy) |
| 3. ____/____/____(dd/mm/yyyy) 8. ____/____/____(dd/mm/yyyy) |
| 4. ____/____/____(dd/mm/yyyy) 9. ____/____/____(dd/mm/yyyy) |
| 5. ____/____/____(dd/mm/yyyy) 10. ____/____/____(dd/mm/yyyy) |
| **Positive *respiratory* culture** (specify organism *Genus species*) at any point during hospitalization |
| 1. ____/____/____(dd/mm/yyyy) 6. ____/____/____(dd/mm/yyyy) |
| 2. ____/____/____(dd/mm/yyyy) 7. ____/____/____(dd/mm/yyyy) |
| 3. ____/____/____(dd/mm/yyyy) 8. ____/____/____(dd/mm/yyyy) |
| 4. ____/____/____(dd/mm/yyyy) 9. ____/____/____(dd/mm/yyyy) |
| 5. ____/____/____(dd/mm/yyyy) 10. ____/____/____(dd/mm/yyyy) |

**ICU Flu Study - Case Report Form .**Note: Day 1 = first day that patient was deemed critically ill

| Assist control Pressure – **ACPC**; Volume **ACVC** / Pressure Support **PS** / High Frequency Oscillation **HFO** / **APRV** / Synchronized Intermittent Mandatory **SIMV** / Non-invasive **NIV**/ Face Mask **FM** / Nasal prongs **NP /** Other **O** (write**)** | **Day 1 Date:**  **___/___/_____**  (dd/mm/yyyy) | **Day 2 Date:**  **___/___/_____** (dd/mm/yyyy) | **Day 3 Date:**  **___/___/_____** (dd/mm/yyyy) | **Day 7 Date:**  **___/___/_____**  (dd/mm/yyyy) | **Day 14 Date:**  **___/___/_____**  (dd/mm/yyyy) | **Day 28 Date:**  **___/___/_____**  (dd/mm/yyyy) |
| --- | --- | --- | --- | --- | --- | --- |
| Ventilatory mode (see codes above) |  |  |  |  |  |  |
| FiO_2_ (0.21-1.0) |  |  |  |  |  |  |
| Tidal volume (use expiratory) ml |  |  |  |  |  |  |
| Set PEEP cmH_2_0 |  |  |  |  |  |  |
| Peak pressure (PEEP + driving Pressure) cmH_2_0 |  |  |  |  |  |  |
| Plateau pressure (if measured) cmH_2_0 |  |  |  |  |  |  |
| Mean airway pressure cmH_2_0 |  |  |  |  |  |  |
| Respiratory rate (total) or Frequency (if HFO) |  |  |  |  |  |  |
| **ABG / Saturation / CXR** (‘’ND” if not done) |  |  |  |  |  |  |
| pH |  |  |  |  |  |  |
| PaCO_2_ mmHg kPa |  |  |  |  |  |  |
| PaO_2_ mmHg kPa |  |  |  |  |  |  |
| HCO_3_ mEq/L |  |  |  |  |  |  |
| Saturation (Pulse oximetry if no ABG) |  |  |  |  |  |  |
| Chest X-ray (number of quadrants/unilat-bilateral) e.g. 1U | 1 2 3 4 U B | 1 2 3 4 U B | 1 2 3 4 U B | 1 2 3 4 U B | 1 2 3 4 U B | 1 2 3 4 U B |
| Barotrauma (pneumothorax/mediastinum) | Yes No | Yes No | Yes No | Yes No | Yes No | Yes No |
| **Organ Dysfunction** (“ND” if not done) |  |  |  |  |  |  |
| Mean Arterial Pressure mmHg |  |  |  |  |  |  |
| Systolic BP mmHg |  |  |  |  |  |  |
| Heart Rate beats per minute |  |  |  |  |  |  |
| Urine Output ml/last 24 hours |  |  |  |  |  |  |
| Temperature degrees C |  |  |  |  |  |  |
| Creatinine level umol/L mg/dL |  |  |  |  |  |  |
| Dialysis (any mode) | Yes No | Yes No | Yes No | Yes No | Yes No | Yes No |
| Dopamine < or = 5 ug/kg/min OR dobutamine OR milrinone OR levosimendan any dose | Yes No | Yes No | Yes No | Yes No | Yes No | Yes No |
| Dopamine 5-15 OR Epi / Norepi < or = 0.1 ug/kg/min OR vasopressin OR metaraminol OR phenylephrine any dose | Yes No | Yes No | Yes No | Yes No | Yes No | Yes No |
| Dopamine > 15 OR Epi / Norepi > 0.1 ug/kg/min | Yes No | Yes No | Yes No | Yes No | Yes No | Yes No |

***All measurements at 08:00 or closest time measured on study days 1, 2, 3, 7, 14, 28***

**ICU Flu Study - Case Report Form** Note: Day 1 = first day that patient was deemed critically ill

|  | **Day 1 Date:**  **___/___/_____**  (dd/mm/yyyy) | **Day 2 Date:**  **___/___/_____** (dd/mm/yyyy) | **Day 3 Date:**  **___/___/_____** (dd/mm/yyyy) | **Day 7 Date:**  **___/___/_____**  (dd/mm/yyyy) | **Day 14 Date:**  **___/___/_____**  (dd/mm/yyyy) | **Day 28 Date:**  **___/___/_____**  (dd/mm/yyyy) |
| --- | --- | --- | --- | --- | --- | --- |
| AST units/L |  |  |  |  |  |  |
| ALT units/L |  |  |  |  |  |  |
| Bilirubin level umol/L mg/dL |  |  |  |  |  |  |
| Platelet count x10^9^/L |  |  |  |  |  |  |
| CK (CPK) units/L |  |  |  |  |  |  |
| Troponin microg/L |  |  |  |  |  |  |
| Albumin g/L |  |  |  |  |  |  |
| Glucose mmol/L |  |  |  |  |  |  |
| White Blood Cell Count x10^9^/L |  |  |  |  |  |  |
| Neutrophil Count x10^9^/L |  |  |  |  |  |  |
| Lymphocyte Count x10^9^/L |  |  |  |  |  |  |
| Coagulation INR *or* PT (sec) |  |  |  |  |  |  |
| Glasgow coma score (see below for calculation) |  |  |  |  |  |  |
| Pupils (**R**=Both Reactive; **F**=Both Fixed) |  |  |  |  |  |  |
| Central Venous Pressure mm Hg |  |  |  |  |  |  |

***All measurements at 08:00 or closest time measured on study days 1, 2, 3, 7, 14, 28***

**Subjective Comments About Course of Illness or Treatment that you believe are Important to Convey:**

**___________________________________________________________________________**

**___________________________________________________________________________**

**___________________________________________________________________________**

**___________________________________________________________________________**

**ICU Flu Study - Case Report Form Severity of Illness (SAPS 2,3; APACHE II, III, IV) for Adults (>17 years of age), PRISM III (<18 years); *complete once in the 1st 24 hrs of ICU Admission***

| **PHYSIOLOGIC VARIABLES** | **Raw Data** |
| --- | --- |
|  |  |
| **Temperature - core (^o^C),** record the number furthest from 38 |  |
| **MAP (mmHg),** record the number furthest from 90 |  |
| **Systolic Blood Pressure (mmHg),** lowest |  |
| **Vasoactive Medications prior to ICU admission** | Yes No |
| **Heart Rate,** record the number furthest from 75 |  |
| **Respiratory Rate,** record the number furthest from 19 |  |
| If FiO_2_ > 0.5 AND intubated, record FiO_2,_ PCO_2,,_ PaO_2_ | **FiO_2_:** |
|  | **PaO_2_:** |
|  | **PCO_2_:** |
| If FiO_2_ < 0.5 OR not intubated, record only PaO_2_ | **PaO_2_:** |
| **Arterial pH,** record the number furthest from 7.4 and corresponding **PCO_2_** | **pH: PCO_2_:** |
| **Serum Na (mmol/L),** record the number furthest from 145 |  |
| **Serum K (mmol/L),** record the number furthest from 3.5 |  |
| **Serum Creatinine **** (µmol/L), select highest value  (mg/dL), select highest value |  |
| **Blood Urea Nitrogen (mg/dL) or Urea (mmol/L),** record number furthest from 0 |  |
| **Urine Output in 24 hours (ml)** |  |
| **Hematocrit** (%), select number furthest from 45.5 |  |
| **WBC (total/mm^3^),** select number furthest from 11.5 |  |
| **Platelets (10x9/L)** (lowest) |  |
| **Serum HCO_3_** (venous mmol/L) – record the number furthest from 26.5 |  |
| **Serum Albumin,** record the lowest number |  |
| **Serum bilirubin,** record the highest number |  |
| **Serum glucose,** record the number furthest from 130 mg/dL or 7.2 mmol/L |  |
| **PT (seconds)** |  |
| **PTT (seconds)** |  |
| **GLASCOW COMA SCALE (circle number)** |  |
| **Eyes Open** Spontaneously | 4 |
| On spoken command | 3 |
| On pain | 2 |
| No response | 1 |
| **Best Motor Response** To spoken command | 6 |
| *To painful stimulus:* Localized pain | 5 |
| Flexion withdrawal | 4 |
| Flexion abnormal | 3 |
| Extension | 2 |
| No response | 1 |
| **Best Verbal Response** |  |
| *(Not on ventilator)* Oriented & converses | 5 |
| Disoriented & converses | 4 |
| Inappropriate words | 3 |
| Incomprehensible sounds | 2 |
| No response | 1 |
| *(On ventilator)* Appears oriented | 5 |
| Questionably oriented | 3 |
| Generally unresponsive | 1 |
| Pupil Reflexes 1 Fixed and 1 Reactive Both Fixed |  |
