# Supplementary material for: The influence of corticosteroid treatment on the outcome of influenza A(H1N1pdm09)-related critical illness
Source: Crit Care. 2016 Mar 30;20:75. doi: 10.1186/s13054-016-1230-8 (PMC4818504; doi:10.1186/s13054-016-1230-8)
Supplement: Supplementary file 4 [file 13054_2016_1230_MOESM2_ESM.docx]

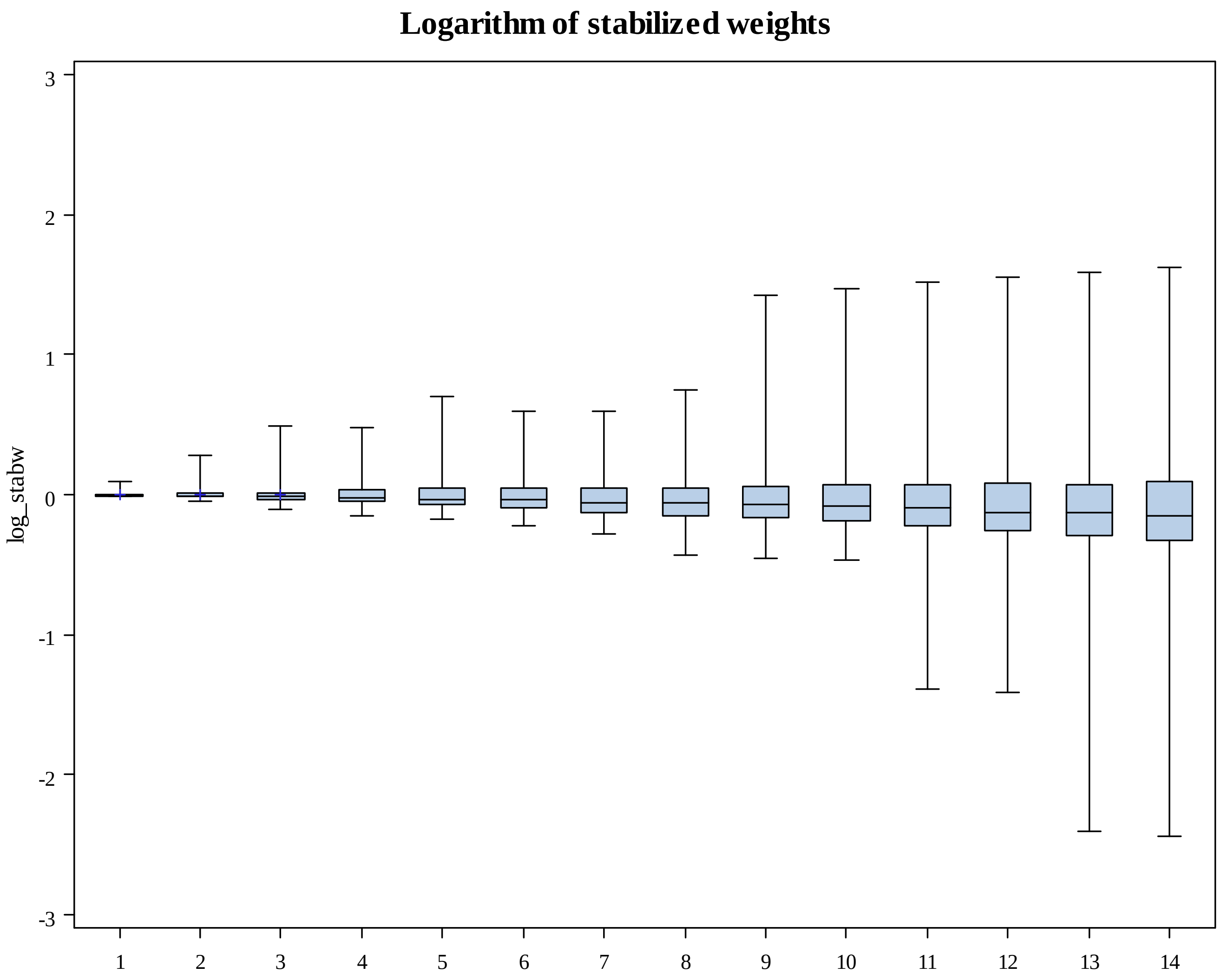
Logarithm of stabilized weights
log_stabw
3
2
1
0
-1
-2
-3
1
2
3
4
5
6
7
8
9
10
11
12
13
14

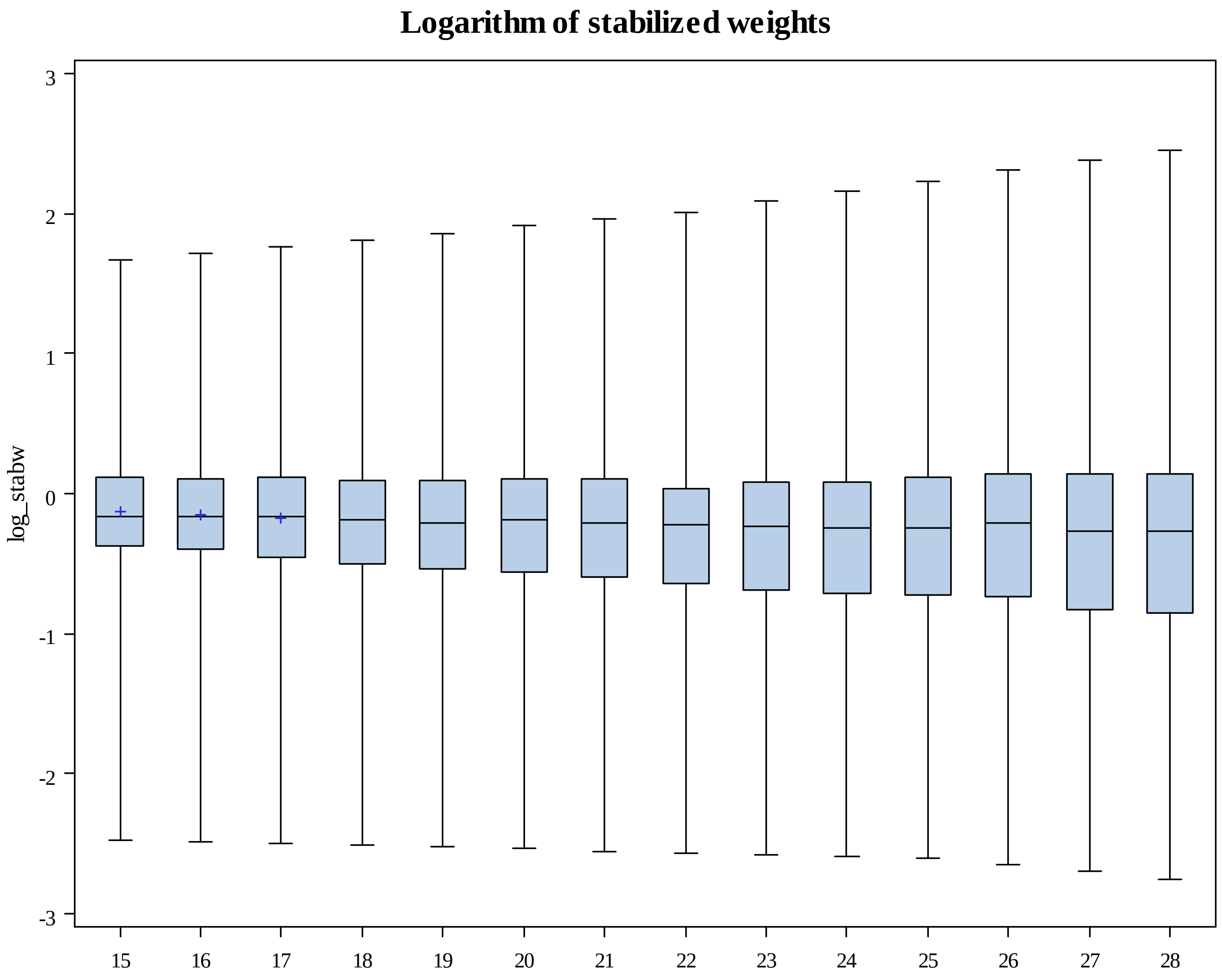
Logarithm of stabilized weights
log_stabw
3
2
1
0
-1
-2
-3
15
16
17
18
19
20
21
22
23
24
25
26
27
28
